# Bat Reovirus as Cause of Acute Respiratory Disease and Encephalitis in Humans, Bangladesh, 2022–2023

**DOI:** 10.3201/eid3112.250797

**Published:** 2025-12

**Authors:** Sharmin Sultana, Ariful Islam, James Ng, Sunil Kumar Dubey, Manjur Hossain Khan, Cheng Guo, Mohammed Ziaur Rahman, Joel M. Montgomery, Syed Moinuddin Satter, Tahmina Shirin, W. Ian Lipkin, Lisa Hensley, Nischay Mishra

**Affiliations:** Institute of Epidemiology, Disease Control and Research (IEDCR), Dhaka, Bangladesh (S. Sultana, A. Islam, M. Hossain Khan, T. Shirin); Gulbali Research Institute, Charles Sturt University, Wagga Wagga, New South Wales, Australia (A. Islam); Center for Infection and Immunity, Mailman School of Public Health, Columbia University, New York, New York, USA (J. Ng, S. Kumar Dubey, C. Guo, W.I. Lipkin, N. Mishra); icddr,b, Dhaka (M.Z. Rahman, S. Moinuddin Satter); Centers for Disease Control and Prevention, Atlanta, Georgia, USA (J.M. Montgomery); Zoonotic and Emerging Disease Research Unit, National Bio and Agro-Defense Facility, USDA Agricultural Research Service, Manhattan, Kansas, USA (L. Hensley)

**Keywords:** Reovirus, Nipah virus, Pteropine orthoreoviruses, viruses, zoonoses, meningitis/encephalitis, bats, Bangladesh

## Abstract

We report 5 patients in Bangladesh presumed to have Nipah virus infections after consuming raw date palm sap. PCR and serology for Nipah virus were negative, but high-throughput sequencing identified *Pteropine orthoreovirus* in archived throat swab samples and virus cultures. This batborne virus should be considered in differential diagnosis of Nipah-like illnesses.

Bats are the natural reservoir of numerous known and novel zoonotic viruses, including rabies, Nipah, Hendra, Marburg, and severe acute respiratory syndrome viruses (*1*). In Bangladesh, Nipah virus (NiV) outbreaks are seasonal, and cases peak during December–April annually. In 2006, the Institute of Epidemiology, Disease Control and Research, Bangladesh (Dhaka, Bangladesh); icddr,b (Dhaka); and US Centers for Disease Control and Prevention (Atlanta, GA, USA) collaboratively established a hospital-based national sentinel surveillance program to address public health risks posed by NiV (*2*). During 2006–2022, the program enrolled >22,000 patients with symptoms of NiV infection. We report detection of *Pteropine orthoreovirus* (PRV) from 5 NiV-negative patients with acute respiratory disease and encephalitis during a 2022–2023 outbreak.

## The Study

PRV is an emerging batborne orthoreovirus previously linked to acute respiratory infections in humans, especially in Southeast Asia (*3**–**5*). PRV is classified under the genus *Orthoreovirus*, family Reoviridae, which includes Nelson Bay virus (NBV), identified in Australia in 1968 (*6*). Zoonotic potential of NBV was confirmed in 2006, when a human case occurred in Melaka, Malaysia (*7*). 

PRVs are nonenveloped, fusogenic viruses with double-stranded RNA genomes composed of 10 segments (S1, S2, S3, S4, M1, M2, M3, L1, L2, and L3). The S1 segment is tricistronic, encoding 3 proteins: cell-attachment protein, fusion-associated small transmembrane protein, and nonstructural protein p17 of unknown function (*8*).

The Bangladesh surveillance program uses quantitative PCR on throat swab samples to test for NiV RNA and on serum for NiV IgG or IgM. During December 2022–March 2023, five patients with presumptive NiV infection diagnoses were admitted to hospitals in Bangladesh but tested NiV-negative (Table). Three patients were admitted to Faridpur Medical College Hospital (MCH) (Faridpur, Bangladesh), and 1 patient each was admitted to Rajshahi MCH (Rajshahi, Bangladesh) and Khulna MCH (Khulna, Bangladesh). All patients had clinical signs and symptoms, including fever, disorientation, altered mental status, abnormal gait, and difficulty breathing. Four patients had a primary diagnosis of encephalitis, and 1 pediatric case had mild symptoms and a primary diagnosis of febrile convulsions (Table). All patients reported consuming raw date palm sap within 2 weeks of symptoms developing. 

**Table:** Characteristics of patients in study of bat reovirus as cause of acute respiratory disease and encephalitis in humans, Bangladesh, 2022–2023*

Geographic and clinical data	Patient identification no.				
	BDB047	BDB051	BDB052	BDB113	BDB040†
Age, y/sex	65/M	17/F	65/M	2/M	56/M
District	Rajbari	Faridpur	Rajbari	Khulna	Sirajganj
Diagnosis at admission	Encephalitis	Encephalitis	Encephalitis	Febrile convulsions	Encephalitis
Symptom onset date	2022 Dec 27	2023 Jan 3	2023 Jan 5	2023 Jan 2	2023 Mar 31
Date palm sap consumption	Y	Y	Y	Y	Y
Frequency, no. times	1	4	1	1	1
Date first consumed	2022 Dec 14	2023 Jan 1	2022 Dec 27	2022 Dec 31	2023 Apr 4
Throat swab collection date	2022 Dec 29	2023 Jan 3	2023 Jan 7	2023 Jan 2	2023 Apr 6
Hospitalization data					
Duration of hospital stay, d	9	2	9	8	14
Symptons at admission					
Fever	Y	Y	Y	Y	Y
Difficulty in breathing	Y	N	N	N	N
Disorientation	Y	Y	Y	N	Y
Vomiting	N	N	Y	N	N
Diarrhea	N	N	Y	N	N
Altered mental status	Y	N	Y	N	Y
Headache	N	Y	Y	N	Y
Stiff neck	Y	N	N	N	Y
Convulsions	N	N	N	Y	N
Unconscious	Y	N	N	Y	N
Salivation	N	N	Y	N	N
Mental status‡	Moderate	Mild	Moderate	Moderate	Mild
Neurologic details, motor	Abnormal gait movements	Mild, abnormal tone	Moderate; abnormal gait, movements	Moderate; abnormal gait, movements	Normal
qPCR and virus culture					
PRV viral load, Ct	24.50	19.17	30.48	33.40	Undetected
Positive virus culture	Y	Y	N	Y	N
Discharge health status	Alive	Alive	Alive	Alive	Alive
Telehealth followup§					
Health status	Alive	Alive	Alive	Alive	Dead
Date of follow	2024 May 21	2024 May 21	2024 May 21	2024 May 21	2024 Oct 30
Condition recorded	Difficulty walking; weakness, disorientation, occasional respiratory distress	Fully recovered; healthy	Generalized weakness and musculoskeletal pain	Fully recovered; healthy	Died in August 2024

*Ct, cycle threshold; PRV, *Pteropine orthoreovirus*. †Patient had prior history of mental disorder and other cognitive disabilities before the onset of Nipah virus–like illness in April 2023. He also consumed date palm sap while hospitalized. The patient later died in August 2024 due to unexplained neurological disease. ‡Moderate: lethargic, decreased response; mild: confused, disoriented, or agitated. §Telehealth followup was conducted >15 mo after hospital discharge.

**Figure 1:**
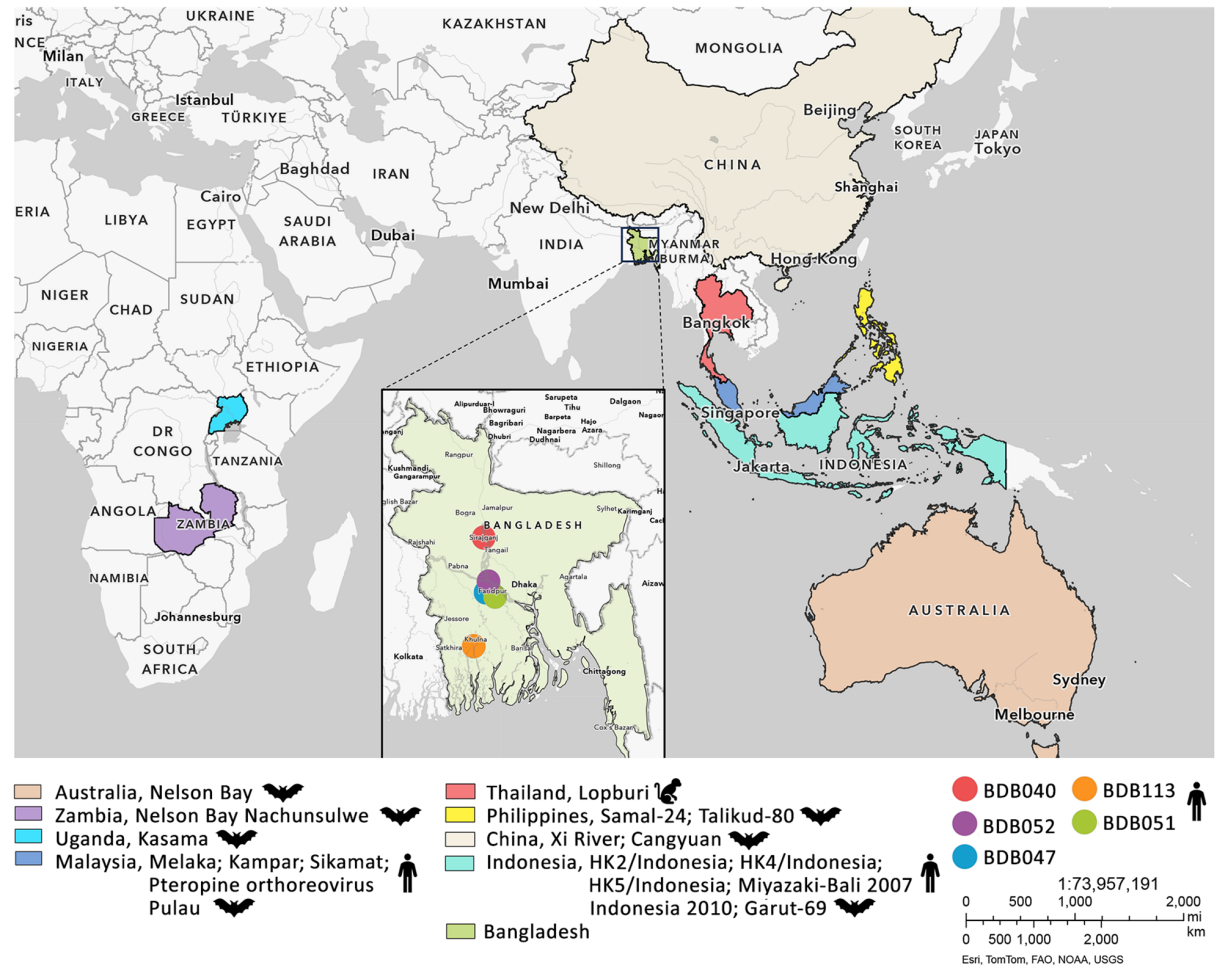
Study locations and locations for related viruses from study of bat reovirus as cause of acute respiratory disease and encephalitis in humans, Bangladesh, 2022–2023. Inset shows Bangladesh with color-coded locations of patients (BDB040, BDB052, BDB047, BDB113, and BDB051) from whom we detected Pteropine orthoreovirus in archived throat swab samples. Larger map shows global locations from which related viruses have been detected in humans, bats, and monkeys.

Patients originated from different geographic regions of Bangladesh. Case-patients BDB047, BDB051, and BDB052 were from Faridpur and Rajbari, within a 30-mile radius of central Bangladesh, near the Padma River Basin (Figure 1). Those 3 patients and pediatric case-patient BDB113 from Khulna (≈180 km south of Faridpur and Rajbari) were hospitalized within the same 2-week period in late December 2022 and early January 2023 (Table). Case-patient BDB040 was admitted in Sirajganj (≈150 km north of Faridpur and Rajbari) during March–April 2023. That patient had a history of chronic mental illness and also consumed raw date palm sap while hospitalized. 

All patients were discharged after 2–3 weeks. During telehealth follow-up >15 months after discharge, case-patients BDB047 and BDB052 reported persistent fatigue, disorientation, and breathing and walking difficulties. Case-patients BDB051 and BDB113 fully recovered, but case-patient BDB040 died in August 2024, following deteriorating health and unexplained neurologic issues (Table).

We conducted viral discovery by using a capture-based agnostic viral sequencing method, VirCapSeq-VERT (VCS) (*9*), on total nucleic acid extracted from archived throat swab samples collected in viral-transport media. We perfomed VCS on NextSeq 2000 (Illumina, https://www.illumina.com), as previously described (*9*). We further used Megablast (MEGA, https://www.megasoftware.net) to compare retrieved sequences to those in GenBank nucleotide databases (Appendix). VCS analysis revealed PRV reads in all patients. We did not identify any other viral or bacterial pathogens in high-throughput sequencing. 

We quantified viral load by using an in-house L2-based quantitative PCR (Appendix Table 1). Case-patient BDB051 had the highest viral load, likely due to the short (≈2-day) interval between raw date palm sap consumption and sample collection (Table). Case-patients BDB047 and BDB052 had higher viral loads than did BDB113 and BDB040.

**Figure 2:**
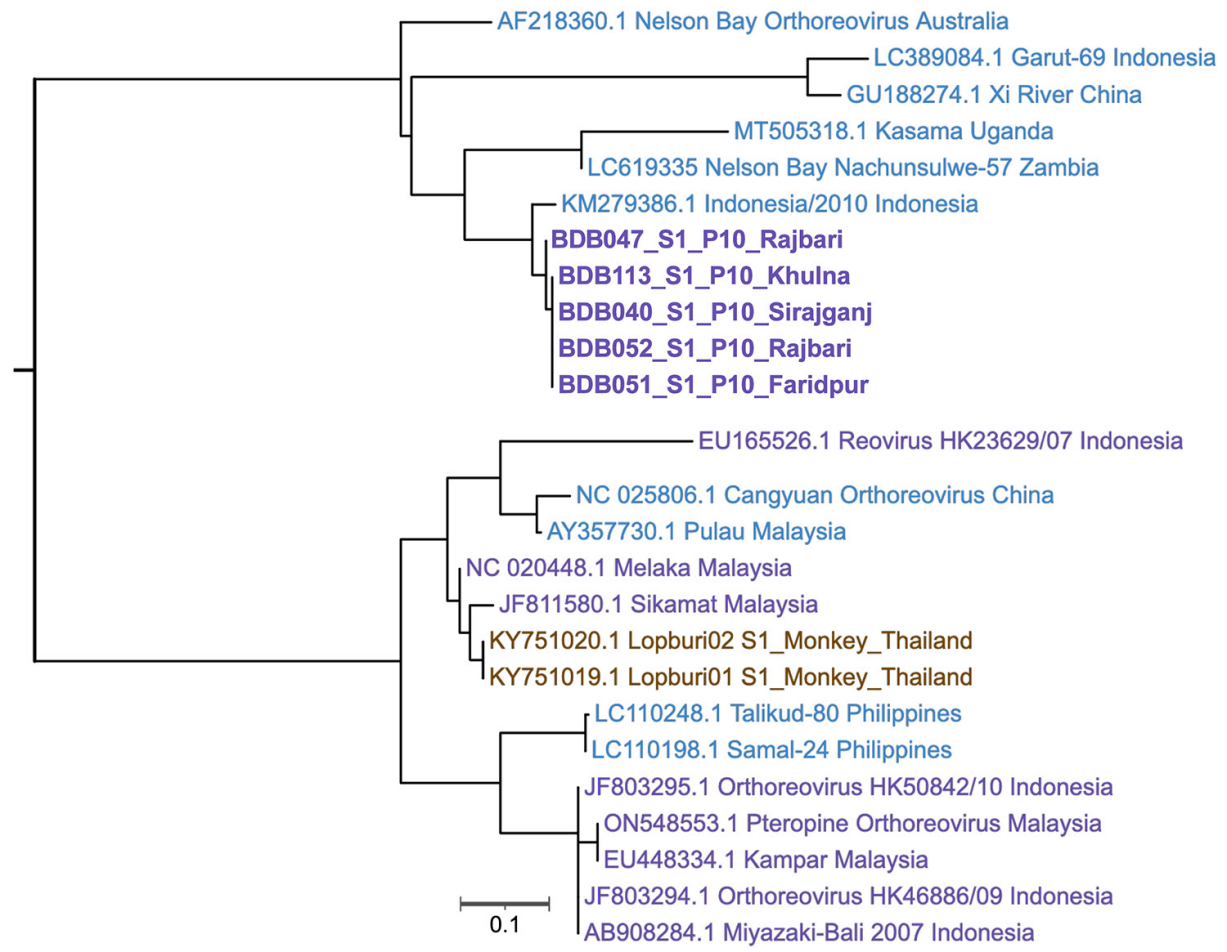
Phylogenic analysis of bat reovirus detected from cases of acute respiratory disease and encephalitis in humans, Bangladesh, 2022–2023. Sequencing of the partial S1 segment showed that Pteropine orthoreovirus from patients in Bangladesh (bold) clustered with 99.3%–100.0% average nucleotide identity (ANI). Bangladesh PRV showed ≈96% ANI with the Indonesia/2010 strain detected from large flying-fox (*Pteropus vampyrus*) in Indonesia (GenBank accession no. KM279386.1) and ≈85% ANI with the Nachunsulwe-57 strain detected from an Egyptian fruit bat (*Rousettus aegyptiacus*) in Zambia (accession no. LC619335) in 2018. GenBank accession numbers are indicated for reference sequences. Scale bar indicates nucleotide substitutions per site.

For phylogenetic analysis, we amplified the partial S1 segment encoding the p10 protein (96 aa) by using a consensus PCR (Figure 2; Appendix Table 2). The Bangladesh PRVs clustered at 99.3%–100.0% average nucleotide identity (ANI). Those PRVs showed ≈96% ANI with the Indonesia/2010 detected from a large flying fox (*Pteropus vampyrus*) in Indonesia, ≈85% ANI with the Nachunsulwe-57 detected from an Egyptian fruit bat (*Rousettus aegyptiacus*) in Zambia, and ≈77% ANI with the Kasama strain detected from an Angolan soft-furred fruit bat (*Lissonycteris angolensis*) in Uganda (*10*,*11*). The Bangladesh PRVs also had >77.0% ANI with Xi River virus from a fulvous fruit bat (*R. leschenaultii*) from China, Garut-69 virus from a large flying fox from Indonesia, and the 1968 prototypic NBV from a grey-headed flying fox (*P. policephalus*) in Australia.

To test whether molecular detection (VCS, quantitative PCR, and partial S1 amplicon sequencing) corresponded to infectious virus presence, we inoculated throat swab samples into MDCK cells and examined for cytopathic effects. After 2 MDCK passages, we passaged PRVs once in Vero cells. We successfully cultured virus from 3 swab samples (case-patients BDB047, BDB051, and BDB113) and sequenced on the MiSeq (Illumina) platform. We mapped reads to PRV genomes by using Geneious Prime (https://www.geneious.com) software. 

**Figure 3:**
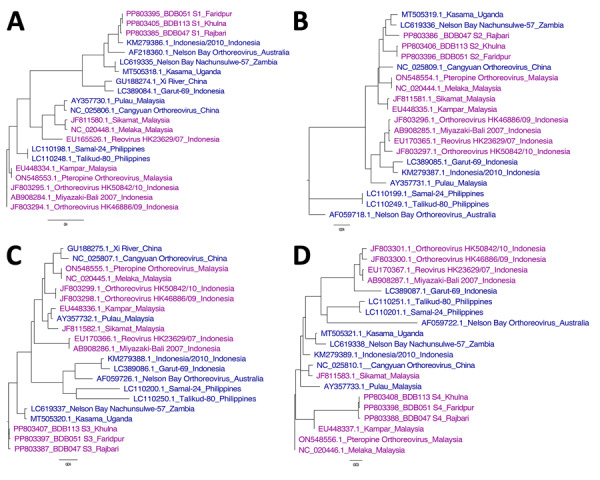
Comparitive phylogeny of bat reovirus as cause of acute respiratory disease and encephalitis in humans, Bangladesh, 2022–2023. A) S1 segment phylogeny showing 96.7%–99.9% average nucleotide identity (ANI) with each other (BDB051, BDB113, and BDB047) and clustered with Indonesia/2010, NBV-Australia, NBV-Nachunsulwe-57, and Kasama virus. B, C) Phylogeny of S2 (B) and S3 (C) segments showing partial consistency with S1 segments. D) S4 segments clustered with Kampar and Melaka strains, previously linked to mild respiratory illness in humans and reported human-to-human transmission. GenBank accession numbers are indicated. Scale bars indicate nucleotide substitutions per site.

Complete coding sequences of all 10 Bangladesh PRV segments (GenBank accession nos. PP803379–408) showed 91.1%–100% ANI among themselves (Appendix Table 3). S1 segments showed 96.7%–99.9% ANI with each other and clustered with Indonesia/2010 strain, NBV-Australia, NBV-Nachunsulwe-57, and Kasama virus (Figure 3, panel A). Phylogeny of S2 and S3 segments were partially consistent with S1 segments (Figure 3, panels B, C). S4 segments clustered with Kampar and Melaka NBV strains (Figure 3, panel D**)**, previously linked to mild respiratory illness in humans and reported human-to-human transmission (*7*,*12*). 

L1, L2, L3, M1, M2, and M3 segments clustered with different PRVs isolated from fruit bats and occasionally from humans in Indonesia and Malaysia (Appendix Figure). That finding suggests unique evolution of each segment from reassortment events among strains circulating in Southeast Asia and long flight ranges of fruit bats. Reassortment is common for segmented RNA virus evolution and enhances risk for zoonotic potential (*13*). All Bangladesh PRV segments showed >76% ANI with NBV-Australia, exceeding the Internationl Committee for Taxonomy of Viruses 2022 species demarcation criteria of <75% ANI (*14*). Thus, the detected PRVs belong to NBV species but are distinct from other mammalian and avian reoviruses.

## Conclusions

Humans in Bangladesh commonly consume raw date palm sap, especially in winter. Raw date palm sap is also a food source for fruit bats during winter and is the primary zoonotic route for NiV spillover from bats to humans (*15*). All 5 patients lived within 30–200 km of central Bangladesh but had no known contact with one another. Patients consumed raw date palm sap within 14 days of symptoms developing. Although no contemporaneous sap samples were available for analysis, we speculate that those PRV infections resulted from the consumption of raw date palm sap contaminated with bat excreta. All 5 patients had severe respiratory and neurologic symptoms, but PRV infections in Malaysia, Indonesia, and Vietnam were associated with milder respiratory disease (*4*,*7*,*12*). Because we focused on severe disease, we cannot exclude the possibility that PRVs in Bangladesh can also cause mild infections.

In summary, human PRV infection can have signs and symptoms similar to those of NiV infection. Like NiV infections, PRV infections can be linked to consumption of date palm sap contaminated with bat excreta. The potential for reassortment in segmented viruses like PRV can result in changes in transmissibility and virulence. Thus, in areas where raw date palm sap is consumed, molecular and serologic surveillance and differential diagnoses of respiratory illnesses with encephalitis and other unexplained febrile illnesses should include PRV, NiV, and other batborne viruses.

Appendix Additional information on bat reovirus as cause of acute respiratory disease and encephalitis in humans, Bangladesh, 2022–2023.
