# Supplementary material for: Bat Reovirus as Cause of Acute Respiratory Disease and Encephalitis in Humans, Bangladesh, 2022–2023
Source: Emerg Infect Dis. 2025 Dec;31(12):2302–7. doi: 10.3201/eid3112.250797 (PMC12782263; doi:10.3201/eid3112.250797)
Supplement: Appendix — Additional information on bat reovirus as cause of acute respiratory disease and encephalitis in humans, Bangladesh, 2022–2023. [file 25-0797-Techapp-1.pdf]

Article DOI: https://doi.org/10.3201/eid3112.250797

EID cannot ensure accessibility for supplementary materials supplied by authors. Readers who have difficulty accessing supplementary content should contact the authors for assistance.

Bat Reovirus as Cause of Acute Respiratory Disease and Encephalitis in Humans, Bangladesh, 2022–2023

Appendix

Nucleic acid extraction and VirCapSeq-VERT library preparation

The NucliSens easyMAG (BioMérieux) was used to extract 250 ul of throat swabs collected in Viral transport media from each patient. Individual VirCapSeq-VERT libraries were prepared using the Hyper Prep kit (KAPA Biosystems, Boston, MA, USA) and unique barcodes (*1*,*2*). RNA extract was DNase treated (DNase I, Ambion, Life Technologies) and first strand cDNA was generated using Superscript III and random hexamer primers for reverse transcription (Invitrogen, Life Technologies). Prior to the second stranded cDNA synthesis, the cDNA was treated with RNase-H using random primer extension and Klenow enzyme (New England Biolabs, Ipswich, MA, USA). Fragmented segments of an average size of 200bp were generated using enzymatic shearing from the resulting double stranded cDNA preparations for HTS (Kapa biosystems, Roche). These fragmented segments were purified using Agencourt Axyprep AMPure Beads (*3*) (Quail 2009) and libraries were prepared using the HyperPlus Library Prep Kit following the manufacturer's protocol. The final libraries were quantified using Agilent TapeStation System. The libraries were then pooled and hybridized with the VirCapSeq-VERT probes set before a final PCR and sequencing on Illumina NextSeq 2000 system.

Data Analytics and Bioinformatics Pipeline

After VirCapSeq-VERT sequencing on Illumina platform, 200bp single end reads were generated, which were de-multiplexed using bcl2fastq software v 1.8.4 into individual samples for further analysis. Illumina adaptor sequences were trimmed from the demultiplexed FastQ

files using cutadapt (v1.8.3) (*4*) (Martin, 2011). Adaptor trimming will be followed by the generation of quality reports using FastQC and filtering with PrinSEQ (*5*). Host background levels will be determined by mapping the filtered reads against the human genome using Bowtie2 mapper (*6*). Assembly of quality filtered and host subtracted reads was performed using MIRA Assembler (v4.0). Assembled contiguous sequences and unique singletons were subjected to homology searches against the entire GenBank nucleotide database using Megablast. All the contiguous sequences and unique singletons from assembly, which did not assign any hit with MegaBLAST, were subjected to NCBI BLASTx. Based on Megablast and BLASTx analysis viral sequences matching with Illumina reads and contigs, were downloaded from NCBI and used for mapping to recover partial or complete genomes and determine genome consensus sequence and breadth of coverage. The FastQ reads were then imported into Geneious Prime (https://www.geneious.com) and the reads were trimmed to remove low quality sequences. The map-to-reference tool was used to assemble the reads using the reference genomes from GenBank.

References

1. Mishra M, Zahra A, Chauhan LV, Thakkar R, Ng J, Joshi S, et al. A short series of case reports of COVID-19 in immunocompromised patients. Viruses. 2022;14:5. PubMed https://doi.org/10.3390/v14050934

2. Briese T, Kapoor A, Mishra N, Jain K, Kumar A, Jabado OJ, et al. Virome capture sequencing enables sensitive viral diagnosis and comprehensive virome analysis. mBio. 2015;6:e01491–15. PubMed https://doi.org/10.1128/mBio.01491-15

3. Quail MA, Swerdlow H, Turner DJ. Improved protocols for the Illumina genome analyzer sequencing system. Curr Protoc Hum Genet. 2009;Chapter 18:182. PubMed https://doi.org/10.1002/0471142905.hg1802s62

4. Martin M. Cutadapt removes adapter sequences from high-throughput sequencing reads [cited 2011 August 2]. EMBnet. 2011;17:3. https://journal.embnet.org/index.php/embnetjournal/article/view/200/458

5. Schmieder R, Edwards R. Quality control and preprocessing of metagenomic datasets. Bioinformatics. 2011;27:863–4. PubMed https://doi.org/10.1093/bioinformatics/btr026

6. Langmead B, Salzberg SL. Fast gapped-read alignment with Bowtie 2. Nat Methods. 2012;9:357–9. PubMed https://doi.org/10.1038/nmeth.1923

Appendix Table 1. L2 segment-based Real-time primers and probe sequences (binding regions are based on Accession # PP803390)

Primers/Probe	Sequence	Binding region
Reo_L2_F	TVTCRTGGCTTGAACCTCGTAG	905–926
Reo_L2_R	CRACTTTCTCYGCCACCTCAGT	1,018–997
Reo_L2_Probe	FAM-CTTCAGGGMTCACGTATGGT-BHQ1	943–962

Appendix Table 2. Partial S1 segment-based Consensus PCR primer sequences for amplification of BD-PRVs (binding regions are based on Accession # PP803395)

Primers	Sequence	Binding region
Reo_S1_p10_F1	TGTCGMTATGAGYGGTGAYTGYGC	20–43
Reo_S1_p10_R1	AMGTSARCTCRCGRMRGCA	393–375
Reo_S1_p10_F2	TGGNAGTGTBCAYTGTCARTC	62–82
Reo_S1_p10_R2	TRKCYRCGAGTYARSGCYAYCA	279–258

Appendix Table 3. Segment-wise (S1, S2, S3, S4, L1, L2, L3, L4, M1, M2, and M3) percentage pairwise nucleotide identities of *Pteropine orthoreoviruses* isolated from Bangladesh (BDB047, BDB051, and BDB113) compared with each other and with available Orthoreovirus sequences from the NCBI database. Corresponding GenBank accession numbers are listed

		Patient sample			GenBank accession number												
S1 segment	Source	BDB047	BDB051	BDB113	AB908284.1_Miyazaki-Bali-2007	AF218360.1_Nelson Bay Australia	AY357730.1_Pulau	EU165526.1_HK2-Indonesia_HK23629/07	EU448334.1_Kampar	GU188274.1_Xi river	JF803294.1_HK4-Indonesia_ HK46886/09	JF803295.1_HK5-Indonesia_ HK50842/10	JF811580.1_ Sikamat/MYS/2010	KM279386.1_ Indonesia/2010	LC110198.1_ Samai-24	LC110248.1_ Talikud-80	LC389084.1_Garut-69
BDB047	Human		96.8	96.7	54.6	76.4	51.5	50.7	53.9	64.4	52.7	52.8	54.4	91.8	53.9	53.9	64.3
BDB051	Human	96.8		99.9	54.7	76.3	50.6	50.5	53.9	64.5	52.7	52.9	53.7	91.5	53.5	53.5	64.3
BDB113	Human	96.7	99.9		54.7	76.4	50.6	50.4	53.9	64.6	52.7	52.9	53.7	91.4	53.5	53.5	64.2
AB908284.1_Miyazaki-Bali-2007	Human	54.6	54.7	54.7		53.6	64.8	64.5	94.6	54.4	99.8	99.4	65.1	55.4	81.3	81.2	54.0
AF218360.1_Nelson_Bay_Australia	Bat	76.4	76.3	76.4	53.6		51.4	50.2	53.5	64.4	52.0	52.1	53.3	76.2	53.6	53.6	64.5
AY357730.1_Pulau	Bat	51.5	50.6	50.6	64.8	51.4		69.7	64.7	51.7	64.1	64.1	79.0	51.0	64.6	64.5	51.0
EU165526.1_HK2-Indonesia_HK23629/07	Human	50.7	50.5	50.4	64.5	50.2	69.7		64.1	51.0	64.6	64.5	70.2	51.5	64.4	64.3	50.0
EU448334.1_Kampar	Human	53.9	53.9	53.9	94.6	53.5	64.7	64.1		53.9	94.5	94.3	64.3	54.5	81.5	81.4	54.2
GU188274.1_Xi river	Bat	64.4	64.5	64.6	54.4	64.4	51.7	51.0	53.9		52.5	52.6	54.6	64.2	53.9	53.9	84.9
JF803294.1_HK4-Indonesia_HK46886/09	Human	52.7	52.7	52.7	99.8	52.0	64.1	64.6	94.5	52.5		99.6	63.3	53.6	80.6	80.6	52.6
JF803295.1_HK5-Indonesia_HK50842/10	Human	52.8	52.9	52.9	99.4	52.1	64.1	64.5	94.3	52.6	99.6		63.4	53.6	80.3	80.2	53.0
JF811580.1_Sikamat/MYS/2010	Human	54.4	53.7	53.7	65.1	53.3	79.0	70.2	64.3	54.6	63.3	63.4		53.2	64.8	64.7	54.2
KM279386.1__Indonesia/2010	Bat	91.8	91.5	91.4	55.4	76.2	51.0	51.5	54.5	64.2	53.6	53.6	53.2		54.3	54.3	63.5
LC110198.1_Samai-24	Bat	53.9	53.5	53.5	81.3	53.6	64.6	64.4	81.5	53.9	80.6	80.3	64.8	54.3		99.9	53.8
LC110248.1_Talikud-80	Bat	53.9	53.5	53.5	81.2	53.6	64.5	64.3	81.4	53.9	80.6	80.2	64.7	54.3	99.9		53.8
LC389084.1_Garut-69	Bat	64.3	64.3	64.2	54.0	64.5	51.0	50.0	54.2	84.9	52.6	53.0	54.2	63.5	53.8	53.8	
LC619335_Nelson_bay_Nachunsulwe-57	Bat	77.3	77.3	77.4	55.3	75.5	51.6	50.0	54.8	64.4	53.5	53.8	54.2	76.4	53.9	53.9	63.7
MT505318.1_Kasama_virus	Bat	75.2	75.1	75.2	53.6	73.6	50.4	48.6	53.2	62.7	52.2	52.5	52.4	74.6	52.4	52.4	62.3
NC_020448.1_Melaka	Human	54.2	53.6	53.6	65.3	52.5	78.7	70.0	64.5	54.2	63.7	63.8	96.0	53.2	65.3	65.2	53.8
NC_025806.1_Cangayun	Bat	52.5	51.9	51.9	66.0	52.4	94.2	70.5	65.8	53.8	64.2	64.2	80.1	52.1	65.8	65.8	52.9
ON548553.1_Pteropine_Malaysia	Human	53.9	54.0	54.0	94.7	53.6	64.7	64.1	99.9	53.9	94.6	94.3	64.3	54.6	81.5	81.5	54.2

S2 Segment		BDB047	BDB051	BDB113	AB908285.1_Miyazaki-Bali-2007	AF059718.1_Nelson_Bay_Australia	AY357731.1_Pulau	EU170365.1_HK2-Indonesia_HK23629/07	EU448335.1_Kampar	JF803296.1_HK4-Indonesia_HK46886/09	JF803297.1_HK5-Indonesia_HK50842/10	JF811581.1_Sikamat/MYS/2010	KM279387.1_Indonesia/2010	LC110199.1_Samai-24	LC110249.1_Talikud-80	LC389085.1_Garut-69	LC619336_Nelson_bay_Nachunsulwe-57
BDB047	Human		93.7	93.6	90.1	84.5	90.4	90.3	93.0	90.2	89.4	92.8	89.0	82.6	82.7	89.0	94.9
BDB051	Human	93.7		99.8	90.5	85.0	90.4	90.5	93.7	90.4	89.5	93.5	89.2	82.5	82.6	88.7	94.3
BDB113	Human	93.6	99.8		90.4	84.8	90.3	90.4	93.6	90.3	89.3	93.3	89.1	82.4	82.4	88.5	94.2
AB908285.1_Miyazaki-Bali-2007	Human	90.1	90.5	90.4		85.8	93.1	99.5	90.2	99.4	97.0	89.9	94.0	83.8	83.8	93.1	89.5
AF059718.1_Nelson_Bay_Australia	Bat	84.5	85.0	84.8	85.8		86.0	85.8	85.2	85.9	86.4	85.2	84.5	88.1	88.2	85.7	84.4
AY357731.1_Pulau	Bat	90.4	90.4	90.3	93.1	86.0		93.1	91.5	93.0	92.5	91.4	91.7	83.1	83.2	90.9	89.9
EU170365.1_HK2-Indonesia_HK23629/07	Human	90.3	90.5	90.4	99.5	85.8	93.1		90.4	99.4	97.0	90.1	94.4	83.8	83.8	93.2	89.5
EU448335.1_Kampar	Human	93.0	93.7	93.6	90.2	85.2	91.5	90.4		90.3	89.8	99.6	89.2	82.7	82.8	89.3	94.0
JF803296.1_HK4-Indonesia_HK46886/09	Human	90.2	90.4	90.3	99.4	85.9	93.0	99.4	90.3		96.9	90.0	94.1	83.7	83.8	93.2	89.6
JF803297.1_HK5-Indonesia_HK50842/10	Human	89.4	89.5	89.3	97.0	86.4	92.5	97.0	89.8	96.9		89.5	93.6	83.8	83.9	93.1	88.7
JF811581.1_Sikamat/MYS/2010	Human	92.8	93.5	93.3	89.9	85.2	91.4	90.1	99.6	90.0	89.5		89.0	82.7	82.8	89.0	93.7
KM279387.1_Indonesia/2010	Bat	89.0	89.2	89.1	94.0	84.5	91.7	94.4	89.2	94.1	93.6	89.0		83.5	83.6	96.8	88.2
LC110199.1_Samai-24	Bat	82.6	82.5	82.4	83.8	88.1	83.1	83.8	82.7	83.7	83.8	82.7	83.5		99.9	84.2	82.2
LC110249.1_Talikud-80	Bat	82.7	82.6	82.4	83.8	88.2	83.2	83.8	82.8	83.8	83.9	82.8	83.6	99.9		84.3	82.3
LC389085.1_Garut-69	Bat	89.0	88.7	88.5	93.1	85.7	90.9	93.2	89.3	93.2	93.1	89.0	96.8	84.2	84.3		88.4
LC619336_Nelson_bay_Nachunsulwe-57	Bat	94.9	94.3	94.2	89.5	84.4	89.9	89.5	94.0	89.6	88.7	93.7	88.2	82.2	82.3	88.4	
MT505319.1_Kasama_virus	Bat	94.5	93.6	93.4	90.8	84.7	91.7	90.8	94.2	90.9	90.3	93.9	89.3	83.2	83.3	89.3	97.1
NC_020444.1_Melaka	Human	93.1	93.5	93.3	90.4	84.9	91.6	90.4	95.1	90.1	89.6	94.9	89.1	82.7	82.7	88.8	93.4
NC_025809.1_Cangyuan	Bat	93.1	93.2	93.1	90.9	85.2	91.2	91.1	95.1	90.9	90.4	94.8	89.7	83.0	83.1	89.5	93.3
ON548554.1_Pteropine_Malaysia	Human	93.1	93.5	93.3	90.4	84.9	91.6	90.4	95.1	90.1	89.6	94.9	89.1	82.7	82.7	88.8	93.4

S3 Segment		BDB047	BDB051	BDB113	AB908286.1_Miyazaki-Bali-2007	AF059726.1_Nelson_Bay_Australia	AY357732.1_Pulau	EU170366.1_HK23629/07	EU448336.1_Kampar	GU188275.1_Xi_River	JF803298.1_HK4-Indonesia_HK46886/09	JF803299.1_HK4-Indonesia_HK50842/10	JF811582.1_Sikamat/MYS/2010	KM279388.1_Indonesia-2010	LC110200.1_Samai-24	LC110250.1_Talikud-80	LC389086.1_Garut-69
BDB047	Human		99.2	99.4	90.0	87.8	91.7	89.1	91.4	91.7	91.6	91.5	91.5	86.0	85.7	85.9	85.8
BDB051	Human	99.2		99.8	89.9	88.0	91.5	88.8	91.3	91.4	91.3	91.2	91.4	85.9	85.8	85.9	85.9
BDB113	Human	99.4	99.8		90.1	87.9	91.8	89.1	91.5	91.6	91.6	91.5	91.6	86.0	85.7	86.0	86.0
AB908286.1_Miyazaki-Bali-2007	Human	90.0	89.9	90.1		87.6	90.7	99.7	90.3	90.7	90.3	90.2	90.5	86.6	85.6	85.1	86.3
AF059726.1_Nelson_Bay_Australia	Bat	87.8	88.0	87.9	87.6		88.1	86.8	87.7	87.3	86.7	86.6	87.5	91.4	87.6	86.6	91.2

		Patient sample			GenBank accession number												
S1 segment	Source	BDB047	BDB051	BDB113	AB908284.1_Miyazaki-Bali-2007	AF218360.1_Nelson Bay Australia	AY357730.1_Pulau	EU165526.1_HK2-Indonesia_HK23629/07	EU448334.1_Kampar	GU188274.1_Xi river	JF803294.1_HK4-Indonesia_HK46886/09	JF803295.1_HK5-Indonesia_HK50842/10	JF811580.1_Sikamat/MYS/2010	KM279386.1_Indonesia/2010	LC110198.1_Samai-24	LC110248.1_Talikud-80	LC389084.1_Garut-69
AY357732.1_Pulau	Bat	91.7	91.5	91.8	90.7	88.1		89.9	98.7	94.0	95.1	95.0	96.9	87.3	86.6	85.6	87.0
EU170366.1_HK23629/07	Human	89.1	88.8	89.1	99.7	86.8	89.9		89.4	89.8	90.1	90.0	89.5	85.9	84.2	83.8	85.3
EU448336.1_Kampar	Human	91.4	91.3	91.5	90.3	87.7	98.7	89.4		93.5	94.5	94.4	96.3	87.1	86.1	85.3	86.6
GU188275.1_Xi_River	Bat	91.7	91.4	91.6	90.7	87.3	94.0	89.8	93.5		94.1	94.0	93.3	86.4	86.5	85.8	86.6
JF803298.1_HK4-Indonesia_HK46886/09	Human	91.6	91.3	91.6	90.3	86.7	95.1	90.1	94.5	94.1		99.9	94.2	85.7	84.9	84.7	85.1
JF803299.1_HK4-Indonesia_HK50842/10	Human	91.5	91.2	91.5	90.2	86.6	95.0	90.0	94.4	94.0	99.9		94.1	85.6	85.0	84.6	85.0
JF811582.1_Sikamat/MYS/2010	Human	91.5	91.4	91.6	90.5	87.5	96.9	89.5	96.3	93.3	94.2	94.1		87.0	86.2	85.6	86.7
KM279388.1_Indonesia-2010	Bat	86.0	85.9	86.0	86.6	91.4	87.3	85.9	87.1	86.4	85.7	85.6	87.0		86.1	84.8	97.6
LC110200.1_Samai-24	Bat	85.7	85.8	85.7	85.6	87.6	86.6	84.2	86.1	86.5	84.9	85.0	86.2	86.1		86.2	86.3
LC110250.1_Talikud-80	Bat	85.9	85.9	86.0	85.1	86.6	85.6	83.8	85.3	85.8	84.7	84.6	85.6	84.8	86.2		84.9
LC389086.1_Garut-69	Bat	85.8	85.9	86.0	86.3	91.2	87.0	85.3	86.6	86.6	85.1	85.0	86.7	97.6	86.3	84.9	
LC619337_Nelson_bay_Nachunsulwe-57	Bat	96.0	95.8	96.1	90.3	87.4	92.2	89.3	92.1	91.4	91.5	91.4	91.8	86.4	85.9	86.0	86.4
MT505320.1_Kasama	Bat	96.0	96.0	96.2	90.4	87.4	92.0	89.6	92.0	91.4	91.6	91.5	91.8	86.5	85.7	86.0	86.4
NC_020445.1_Melaka	Human	91.3	91.1	91.2	90.4	86.8	94.6	89.2	94.4	96.2	94.3	94.2	94.0	85.9	86.5	85.6	85.9
NC_025807.1_Cangyuan	Bat	91.1	90.8	91.0	89.7	86.5	93.1	88.6	92.6	97.9	93.2	93.1	92.8	85.7	86.4	85.3	86.2
ON548555.1_Pteropine_Malaysia	Human	91.4	91.2	91.3	90.4	86.9	94.5	89.3	94.3	96.3	94.3	94.3	94.0	85.9	86.6	85.7	85.9
S4 Segment		BDB047	BDB051	BDB113	AB908287.1_Miyazaki-Bali-2007	AF059722.1_Nelson_Bay_Australia	AY357733.1_Pulau	EU170367.1_HK2-Indonesia	EU448337.1_Kampar	JF803300.1_HK4-Indonesia	JF803301.1_HK4-Indonesia	JF811583.1_Sikamat/MYS/2010	KM279389.1_Indonesia/2010	LC110201.1_Samai-24	LC110251.1_Talikud-80	LC389087.1_Garut-69	LC619338_Nelson_bay_Nachunsulwe-57
BDB047	Human		99.3	99.3	87.8	81.3	86.4	87.5	91.4	87.4	86.9	87.5	88.6	86.2	86.3	86.4	87.5
BDB051	Human	99.3		99.8	88.0	81.3	86.5	87.7	91.4	87.6	87.1	87.8	88.7	86.5	86.5	86.5	87.6
BDB113	Human	99.3	99.8		87.8	81.1	86.5	87.5	91.4	87.4	86.9	87.8	88.8	86.5	86.5	86.5	87.8
AB908287.1_Miyazaki-Bali-2007	Human	87.8	88.0	87.8		82.8	85.6	99.6	88.2	97.6	97.7	87.6	87.9	86.2	86.2	92.6	86.2
AF059722.1_Nelson_Bay_Australia	Bat	81.3	81.3	81.1	82.8		81.7	82.7	82.9	82.5	82.6	82.8	82.5	82.9	82.6	81.7	83.0
AY357733.1_Pulau	Bat	86.4	86.5	86.5	85.6	81.7		85.5	91.6	85.8	85.3	93.3	93.5	86.4	85.8	84.4	91.1
EU170367.1_HK2-Indonesia	Human	87.5	87.7	87.5	99.6	82.7	85.5		88.0	97.5	97.8	87.5	87.9	86.0	85.9	92.5	86.0
EU448337.1_Kampar	Human	91.4	91.4	91.4	88.2	82.9	91.6	88.0		88.0	87.9	92.7	94.0	87.9	87.2	86.4	92.0
JF803300.1_HK4-Indonesia	Human	87.4	87.6	87.4	97.6	82.5	85.8	97.5	88.0		97.9	87.1	87.7	86.2	86.3	92.3	86.2
JF803301.1_HK4-Indonesia	Human	86.9	87.1	86.9	97.7	82.6	85.3	97.8	87.9	97.9		87.0	87.8	85.8	85.9	92.1	86.1
JF811583.1_Sikamat/MYS/2010	Human	87.5	87.8	87.8	87.6	82.8	93.3	87.5	92.7	87.1	87.0		95.2	88.0	87.5	86.1	92.3
KM279389.1_Indonesia/2010	Bat	88.6	88.7	88.8	87.9	82.5	93.5	87.9	94.0	87.7	87.8	95.2		88.4	87.8	85.9	94.1
LC110201.1_Samai-24	Bat	86.2	86.5	86.5	86.2	82.9	86.4	86.0	87.9	86.2	85.8	88.0	88.4		98.5	85.6	87.8
LC110251.1_Talikud-80	Bat	86.3	86.5	86.5	86.2	82.6	85.8	85.9	87.2	86.3	85.9	87.5	87.8	98.5		85.3	87.0
LC389087.1_Garut-69	Bat	86.4	86.5	86.5	92.6	81.7	84.4	92.5	86.4	92.3	92.1	86.1	85.9	85.6	85.3		84.9
LC619338_Nelson_bay_Nachunsulwe-57	Bat	87.5	87.6	87.8	86.2	83.0	91.1	86.0	92.0	86.2	86.1	92.3	94.1	87.8	87.0	84.9	
MT505321.1_Kasama_virus	Bat	87.7	87.8	87.8	87.3	82.9	92.5	87.2	92.6	87.3	87.2	93.7	95.1	88.5	87.6	85.3	97.4
NC_020446.1_Melaka	Human	88.6	88.7	88.7	88.0	83.3	94.7	88.0	95.8	88.0	87.6	96.0	96.3	87.9	87.3	86.3	93.5
NC_025810.1_Cangayun	Bat	88.0	88.2	88.2	87.9	82.8	93.3	87.8	92.5	87.5	87.4	98.2	95.0	88.3	87.6	86.1	92.2
ON548556.1_Pteropine_Malaysia	Human	88.6	88.7	88.7	88.0	83.3	94.7	88.0	95.8	88.0	87.6	96.0	96.3	87.9	87.3	86.3	93.5
L1 Segment		BDB047	BDB051	BDB113	AB908278.1_Miyazaki-Bali-2007	JF342654.1_Kampar	JF342666.1_Pulau	JF342672.1_Nelson_Bay-Australia	KM279380.1_Indonesia-2010	LC110205.1_Samai-24	LC110255.1_Talikud-80	LC389091.1_Garut-69	LC619329_Nelson_Bay_Nachunsulwe-57	MT505322.1_Kasama	NC_020439.1_Melaka	NC_025801.1_Cangyuan	ON548560.1_Pteropine_Malaysia
BDB047	Human		98.0	97.9	89.9	89.4	90.4	81.0	89.7	81.2	81.3	80.8	95.2	95.2	89.5	89.6	89.6
BDB051	Human	98.0		99.8	89.7	89.4	90.4	81.0	89.8	81.2	81.3	80.9	95.0	95.0	89.7	89.6	89.7
BDB113	Human	97.9	99.8		89.6	89.4	90.4	81.0	89.7	81.1	81.3	80.9	95.0	95.0	89.7	89.6	89.7
AB908278.1_Miyazaki-Bali-2007	Human	89.9	89.7	89.6		91.1	92.0	81.8	91.0	82.1	82.4	81.7	90.5	90.6	91.6	91.1	91.6
JF342654.1_Kampar	Human	89.4	89.4	89.4	91.1		97.3	82.0	94.9	82.2	82.7	81.9	90.2	90.3	98.0	93.1	98.1
JF342666.1_Pulau	Bat	90.4	90.4	90.4	92.0	97.3		81.8	96.1	82.6	83.0	81.8	91.3	91.3	97.9	94.2	97.9
JF342672.1_Nelson_Bay-Australia	Bat	81.0	81.0	81.0	81.8	82.0	81.8		81.4	81.0	80.7	80.0	81.5	81.5	81.8	81.4	81.9
KM279380.1_Indonesia-2010	Bat	89.7	89.8	89.7	91.0	94.9	96.1	81.4		82.5	82.3	81.2	90.5	90.5	95.4	93.1	95.4
LC110205.1_Samai-24	Bat	81.2	81.2	81.1	82.1	82.2	82.6	81.0	82.5		88.5	81.0	81.8	81.7	82.4	81.7	82.5
LC110255.1_Talikud-80	Bat	81.3	81.3	81.3	82.4	82.7	83.0	80.7	82.3	88.5		81.4	81.7	81.6	82.6	82.2	82.6
LC389091.1_Garut-69	Bat	80.8	80.9	80.9	81.7	81.9	81.8	80.0	81.2	81.0	81.4		81.5	81.3	81.5	81.7	81.5
LC619329_Nelson_Bay_Nachunsulwe-57	Bat	95.2	95.0	95.0	90.5	90.2	91.3	81.5	90.5	81.8	81.7	81.5		99.3	90.5	90.3	90.5
MT505322.1_Kasama	Bat	95.2	95.0	95.0	90.6	90.3	91.3	81.5	90.5	81.7	81.6	81.3	99.3		90.6	90.4	90.6
NC_020439.1_Melaka	Human	89.5	89.7	89.7	91.6	98.0	97.9	81.8	95.4	82.4	82.6	81.5	90.5	90.6		93.4	99.8
NC_025801.1_Cangyuan	Bat	89.6	89.6	89.6	91.1	93.1	94.2	81.4	93.1	81.7	82.2	81.7	90.3	90.4	93.4		93.4
ON548560.1_Pteropine_Malaysia	Human	89.6	89.7	89.7	91.6	98.1	97.9	81.9	95.4	82.5	82.6	81.5	90.5	90.6	99.8	93.4	

		Patient sample			GenBank accession number												
S1 segment	Source	BDB047	BDB051	BDB113	AB908284.1_Miyazaki-Bali-2007	AF218360.1_Nelson Bay Australia	AY357730.1_Pulau	EU165526.1_HK2-Indonesia_HK23629/07	EU448334.1_Kampar	GU188274.1_Xi river	JF803294.1_HK4-Indonesia_HK46886/09	JF803295.1_HK5-Indonesia_HK50842/10	JF811580.1_Sikamat/MYS/2010	KM279386.1_Indonesia/2010	LC110198.1_Samai-24	LC110248.1_Talikud-80	LC389084.1_Garut-69
L2 Segment		BDB047	BDB051	BDB113	AB908279.1_Miyazaki-Bali-2007	JF342655.1_Kampar	JF342667.1_Pulau	JF342673.1_Nelson_Bay-Australia	KM279381.1_Indonesia-2010	LC110206.1_Samai-24	LC110256.1_Talikud-80	LC389092.1_Garut-69	LC619330_Nelson_Bay_Nachunsulwe-57	MT505323.1_Kasama	NC_020447.1_Melaka	NC_025802.1_Cangyuan	ON548561.1_Pteropine_Malaysia
BDB047	Human		91.1	91.1	87.7	87.0	87.1	82.8	86.1	85.4	85.4	86.6	90.9	90.7	87.0	86.6	87.1
BDB051	Human	91.1		99.9	87.4	87.0	87.1	82.9	86.1	85.4	85.4	86.0	91.9	91.8	87.0	86.7	87.0
BDB113	Human	91.1	99.9		87.5	86.9	87.0	83.0	86.1	85.4	85.4	86.0	91.9	91.8	87.0	86.6	87.0
AB908279.1_Miyazaki-Bali-2007	Human	87.7	87.4	87.5		88.9	89.0	83.5	88.7	86.0	85.9	88.7	87.1	87.0	89.0	88.5	88.9
JF342655.1_Kampar	Human	87.0	87.0	86.9	88.9		94.6	83.5	93.2	85.4	85.6	92.4	86.7	86.5	98.6	97.9	98.7
JF342667.1_Pulau	Bat	87.1	87.1	87.0	89.0	94.6		83.5	94.8	85.5	85.7	94.0	87.0	86.8	94.6	94.0	94.6
JF342673.1_Nelson_Bay-Australia	Bat	82.8	82.9	83.0	83.5	83.5	83.5		83.3	83.7	83.9	83.9	83.6	83.5	83.7	83.7	83.7
KM279381.1_Indonesia-2010	Bat	86.1	86.1	86.1	88.7	93.2	94.8	83.3		85.0	85.4	97.8	86.6	86.2	93.3	92.8	93.2
LC110206.1_Samai-24	Bat	85.4	85.4	85.4	86.0	85.4	85.5	83.7	85.0		96.8	85.3	85.2	84.9	85.3	85.0	85.3
LC110256.1_Talikud-80	Bat	85.4	85.4	85.4	85.9	85.6	85.7	83.9	85.4	96.8		85.7	85.4	85.1	85.6	85.4	85.6
LC389092.1_Garut-69	Bat	86.6	86.0	86.0	88.7	92.4	94.0	83.9	97.8	85.3	85.7		86.5	86.2	92.5	92.2	92.5
LC619330_Nelson_Bay_Nachunsulwe-57	Bat	90.9	91.9	91.9	87.1	86.7	87.0	83.6	86.6	85.2	85.4	86.5		99.2	86.7	86.4	86.7
MT505323.1_Kasama	Bat	90.7	91.8	91.8	87.0	86.5	86.8	83.5	86.2	84.9	85.1	86.2	99.2		86.4	86.2	86.4
NC_020447.1_Melaka	Human	87.0	87.0	87.0	89.0	98.6	94.6	83.7	93.3	85.3	85.6	92.5	86.7	86.4		97.9	99.8
NC_025802.1_Cangyuan	Bat	86.6	86.7	86.6	88.5	97.9	94.0	83.7	92.8	85.0	85.4	92.2	86.4	86.2	97.9		98.0
ON548561.1_Pteropine_Malaysia	Human	87.1	87.0	87.0	88.9	98.7	94.6	83.7	93.2	85.3	85.6	92.5	86.7	86.4	99.8	98.0	
L3 Segment		BDB047	BDB051	BDB113	JF342656.1_Kampar	AB908280.1_Miyazaki-Bali-2007	JF342668.1_Pulau	JF342674.1_Nelson_Bay	KM279382.1_Indonesia-2010	LC110207.1_Samai-24	LC110257.1_Talikud-80	LC389093.1_Garut-69	LC619331_Nelson_Bay_Nachunsulwe-57	MT505324.1_Kasama	NC_020440.1_Melaka	NC_025803.1_Cangyuan	ON548562.1_Pteropine_Malaysia
BDB047	Human		99.6	99.5	93.2	88.3	91.2	85.0	84.5	86.5	86.3	84.2	94.6	82.2	93.0	92.6	93.1
BDB051	Human	99.6		99.9	93.1	88.4	91.3	85.0	84.4	86.5	86.4	84.2	94.6	82.2	93.1	92.7	93.2
BDB113	Human	99.5	99.9		93.3	88.4	91.4	85.0	84.5	86.6	86.5	84.3	94.6	82.1	93.3	92.9	93.3
JF342656.1_Kampar	Human	93.2	93.1	93.3		89.6	93.1	85.5	85.1	88.0	87.8	85.0	93.6	81.0	95.3	94.7	95.3
AB908280.1_Miyazaki-Bali-2010	Human	88.3	88.4	88.4	89.6		89.8	84.4	84.6	87.2	87.2	84.5	89.1	77.2	89.4	89.5	89.5
JF342668.1_Pulau	Bat	91.2	91.3	91.4	93.1	89.8		86.0	85.6	88.0	87.7	85.4	91.4	79.1	93.6	93.0	93.6
JF342674.1_Nelson_Bay	Bat	85.0	85.0	85.0	85.5	84.4	86.0		84.5	84.5	84.2	84.3	85.0	73.3	86.1	85.9	86.2
KM279382.1_Indonesia-2010	Bat	84.5	84.4	84.5	85.1	84.6	85.6	84.5		84.7	84.8	97.2	84.6	72.9	85.3	84.5	85.3
LC110207.1_Samai-24	Bat	86.5	86.5	86.6	88.0	87.2	88.0	84.5	84.7		98.8	84.2	86.7	75.0	88.0	87.9	88.0
LC110257.1_Talikud-80	Bat	86.3	86.4	86.5	87.8	87.2	87.7	84.2	84.8	98.8		84.5	86.5	74.9	87.7	87.7	87.8
LC389093.1_Garut-69	Bat	84.2	84.2	84.3	85.0	84.5	85.4	84.3	97.2	84.2	84.5		84.6	73.5	85.2	84.7	85.3
LC619331_Nelson_Bay_Nachunsulwe-57	Bat	94.6	94.6	94.6	93.6	89.1	91.4	85.0	84.6	86.7	86.5	84.6		85.8	93.5	93.1	93.6
MT505324.1_Kasama	Bat	82.2	82.2	82.1	81.0	77.2	79.1	73.3	72.9	75.0	74.9	73.5	85.8		81.0	80.6	81.0
NC_020440.1_Melaka	Human	93.0	93.1	93.3	95.3	89.4	93.6	86.1	85.3	88.0	87.7	85.2	93.5	81.0		97.6	100.0
NC_025803.1_Cangyuan	Bat	92.6	92.7	92.9	94.7	89.5	93.0	85.9	84.5	87.9	87.7	84.7	93.1	80.6	97.6		97.6
ON548562.1_Pteropine_Malaysia	Human	93.1	93.2	93.3	95.3	89.5	93.6	86.2	85.3	88.0	87.8	85.3	93.6	81.0	100.0	97.6	
M1 Segment		BDB047	BDB051	BDB113	AB908281.1_Miyazaki-Bali-2007	JF342657.1_Kampar	JF342669.1_Pulau	JF342675.1_Nelson_Bay	KM279383.1_Indonesia-2010	LC110202.1_Samai-24	LC110252.1_Talikud-80	LC389088.1_Garut-69	LC619332_Nelson_Bay_Nachunsulwe-57	MT505315.1_Kasama	NC_020441.1_Melaka	NC_025804.1_Cangyuan	ON548557.1_Pteropine_Malaysia
BDB047	Human		99.3	99.3	85.1	93.4	91.3	82.1	84.7	81.9	82.2	85.2	94.6	94.9	93.1	97.6	93.1
BDB051	Human	99.3		99.9	85.4	93.6	91.5	82.4	84.9	82.2	82.5	85.2	94.7	94.9	93.4	97.6	93.4
BDB113	Human	99.3	99.9		85.4	93.6	91.5	82.3	84.9	82.2	82.5	85.2	94.7	94.9	93.4	97.6	93.4
AB908281.1_Miyazaki-Bali-2007	Human	85.1	85.4	85.4		86.6	86.9	83.1	94.3	82.0	82.4	92.4	85.1	85.5	86.4	85.7	86.4
JF342657.1_Kampar	Human	93.4	93.6	93.6	86.6		92.6	82.9	86.0	82.4	82.8	86.2	93.6	93.9	98.8	94.1	98.8
JF342669.1_Pulau	Bat	91.3	91.5	91.5	86.9	92.6		82.9	86.2	82.7	82.9	86.3	91.1	91.5	92.4	91.5	92.4
JF342675.1_Nelson_Bay	Bat	82.1	82.4	82.3	83.1	82.9	82.9		83.5	82.7	82.4	82.3	82.2	82.3	82.8	82.2	82.8
KM279383.1_Indonesia-2010	Bat	84.7	84.9	84.9	94.3	86.0	86.2	83.5		82.3	82.5	91.9	85.0	85.1	85.8	85.5	85.8
LC110202.1_Samai-24	Bat	81.9	82.2	82.2	82.0	82.4	82.7	82.7	82.3		98.4	82.0	82.8	82.7	82.1	82.1	82.1
LC110252.1_Talikud-80	Bat	82.2	82.5	82.5	82.4	82.8	82.9	82.4	82.5	98.4		82.2	82.9	82.9	82.5	82.5	82.5
LC389088.1_Garut-69	Bat	85.2	85.2	85.2	92.4	86.2	86.3	82.3	91.9	82.0	82.2		84.6	84.8	86.2	85.4	86.2
LC619332_Nelson_Bay_Nachunsulwe-57	Bat	94.6	94.7	94.7	85.1	93.6	91.1	82.2	85.0	82.8	82.9	84.6		99.2	93.3	95.0	93.3
MT505315.1_Kasama	Bat	94.9	94.9	94.9	85.5	93.9	91.5	82.3	85.1	82.7	82.9	84.8	99.2		93.6	95.4	93.6
NC_020441.1_Melaka	Human	93.1	93.4	93.4	86.4	98.8	92.4	82.8	85.8	82.1	82.5	86.2	93.3	93.6		93.9	100.0
NC_025804.1_Cangyuan	Bat	97.6	97.6	97.6	85.7	94.1	91.5	82.2	85.5	82.1	82.5	85.4	95.0	95.4	93.9		93.9
ON548557.1_Pteropine_Malaysia	Human	93.1	93.4	93.4	86.4	98.8	92.4	82.8	85.8	82.1	82.5	86.2	93.3	93.6	100.0	93.9	

		Patient sample			GenBank accession number												
S1 segment	Source	BDB047	BDB051	BDB113	AB908284.1_Miyazaki-Bali-2007	AF218360.1_Nelson Bay Australia	AY357730.1_Pulau	EU165526.1_HK2-Indonesia_HK23629/07	EU448334.1_Kampar	GU188274.1_Xi river	JF803294.1_HK4-Indonesia_HK46886/09	JF803295.1_HK5-Indonesia_HK50842/10	JF811580.1_Sikamat/MYS/2010	KM279386.1_Indonesia/2010	LC110198.1_Samai-24	LC110248.1_Talikud-80	LC389084.1_Garut-69
M2 Segment		BDB047	BDB051	BDB113	AB908282.1_Miyazaki-Bali-2007	JF342658.1_Kampar	JF342670.1_Pulau	JF342676.1_Nelson_Bay_Australia	KM279384.1_Indonesia-2010	LC110203.1_Samai-24	LC110253.1_Talikud-80	LC389089.1_Garut-69	LC619333_Nelson_Bay_Nachunsulwe-57	MT505316.1_Kasama	NC_020442.1_Melaka	NC_025808.1_Cangyuan	ON548558.1_Pteropine_Malaysia
BDB047	Human		99.8	99.7	79.5	94.1	79.3	79.2	78.9	87.9	78.9	90.5	93.6	93.8	79.2	95.7	79.1
BDB051	Human	99.8		99.9	79.6	94.2	79.4	79.1	79.0	88.0	78.8	90.6	93.7	93.9	79.4	95.8	79.4
BDB113	Human	99.7	99.9		79.6	94.1	79.4	79.0	79.0	88.1	78.9	90.6	93.6	93.8	79.4	95.7	79.3
AB908282.1_Miyazaki-Bali-2007	Human	79.5	79.6	79.6		79.0	92.2	79.9	97.1	79.0	86.5	78.4	78.4	78.1	91.9	79.2	91.8
JF342658.1_Kampar	Human	94.1	94.2	94.1	79.0		78.8	78.8	78.2	88.0	79.1	91.5	95.1	95.0	78.9	95.0	78.8
JF342670.1_Pulau	Bat	79.3	79.4	79.4	92.2	78.8		79.7	92.0	79.8	86.5	79.0	78.7	78.1	94.2	79.2	94.2
JF342676.1_Nelson_Bay_Australia	Bat	79.2	79.1	79.0	79.9	78.8	79.7		78.9	78.6	80.1	77.9	77.9	77.7	79.7	78.1	79.6
KM279384.1_Indonesia-2010	Bat	78.9	79.0	79.0	97.1	78.2	92.0	78.9		78.4	85.1	77.7	77.8	77.9	91.3	78.6	91.4
LC110203.1_Samai-24	Bat	87.9	88.0	88.1	79.0	88.0	79.8	78.6	78.4		79.1	87.0	87.2	87.2	79.3	87.6	79.2
LC110253.1_Talikud-80	Bat	78.9	78.8	78.9	86.5	79.1	86.5	80.1	85.1	79.1		78.9	78.7	78.2	86.2	79.2	86.1
LC389089.1_Garut-69	Bat	90.5	90.6	90.6	78.4	91.5	79.0	77.9	77.7	87.0	78.9		91.6	91.1	78.8	91.4	78.7
LC619333_Nelson_Bay_Nachunsulwe-57	Bat	93.6	93.7	93.6	78.4	95.1	78.7	77.9	77.8	87.2	78.7	91.6		99.0	78.7	94.2	78.6
MT505316.1_Kasama	Bat	93.8	93.9	93.8	78.1	95.0	78.1	77.7	77.9	87.2	78.2	91.1	99.0		78.2	94.3	78.1
NC_020442.1_Melaka	Human	79.2	79.4	79.4	91.9	78.9	94.2	79.7	91.3	79.3	86.2	78.8	78.7	78.2		79.2	99.9
NC_025808.1_Cangyuan	Bat	95.7	95.8	95.7	79.2	95.0	79.2	78.1	78.6	87.6	79.2	91.4	94.2	94.3	79.2		79.1
ON548558.1_Pteropine_Malaysia	Human	79.1	79.4	79.3	91.8	78.8	94.2	79.6	91.4	79.2	86.1	78.7	78.6	78.1	99.9	79.1	
M3 Segment		BDB047	BDB051	BDB113	AB908283.1_Miyazaki-Bali-2007	JF342659.1_Kampar	JF342671.1_Pulau	JF342677.1_Nelson_Bay_Australia	KM279385.1_Indonesia-2010	LC110204.1_Samai-24	LC110254.1_Talikud-80	LC389090.1_Garut-69	LC619334_Nelson_Bay_Nachunsulwe-57	MT505317.1_Kasama	NC_020443.1_Melaka	NC_025805.1_Cangyuan	ON548559.1_Pteropine_Malaysia
BDB047	Human		97.9	97.4	90.8	93.8	93.5	82.6	90.8	83.4	81.4	90.2	96.3	94.8	93.6	92.3	93.8
BDB051	Human	97.9		99.5	90.9	93.6	93.3	82.2	91.0	83.5	80.9	90.2	96.1	94.5	93.5	91.9	93.6
BDB113	Human	97.4	99.5		90.5	93.1	92.8	81.8	90.8	83.2	80.5	89.9	95.7	94.1	93.0	91.4	93.1
AB908283.1_Miyazaki-Bali-2007	Human	90.8	90.9	90.5		93.0	92.8	82.4	94.4	82.9	81.6	96.6	91.1	90.1	92.8	91.5	93.0
JF342659.1_Kampar	Human	93.8	93.6	93.1	93.0		96.2	82.5	91.6	83.4	81.9	91.6	93.7	92.9	98.8	93.7	98.9
JF342671.1_Pulau	Bat	93.5	93.3	92.8	92.8	96.2		82.9	91.4	83.5	82.0	91.7	93.5	92.3	96.0	93.8	96.2
JF342677.1_Nelson_Bay_Australia	Bat	82.6	82.2	81.8	82.4	82.5	82.9		82.2	86.5	84.8	82.5	81.8	81.3	82.8	82.4	82.9
KM279385.1_Indonesia-2010	Bat	90.8	91.0	90.8	94.4	91.6	91.4	82.2		82.1	80.5	93.4	90.5	89.6	91.6	90.7	91.8
LC110204.1_Samai-24	Bat	83.4	83.5	83.2	82.9	83.4	83.5	86.5	82.1		83.4	82.8	82.7	82.2	83.5	82.8	83.6
LC110254.1_Talikud-80	Bat	81.4	80.9	80.5	81.6	81.9	82.0	84.8	80.5	83.4		81.2	81.0	80.9	81.9	81.8	82.0
LC389090.1_Garut-69	Bat	90.2	90.2	89.9	96.6	91.6	91.7	82.5	93.4	82.8	81.2		90.1	89.2	91.7	90.8	91.8
LC619334_Nelson_Bay_Nachunsulwe-57	Bat	96.3	96.1	95.7	91.1	93.7	93.5	81.8	90.5	82.7	81.0	90.1		97.7	93.6	92.4	93.7
MT505317.1_Kasama	Bat	94.8	94.5	94.1	90.1	92.9	92.3	81.3	89.6	82.2	80.9	89.2	97.7		92.7	91.1	92.9
NC_020443.1_Melaka	Human	93.6	93.5	93.0	92.8	98.8	96.0	82.8	91.6	83.5	81.9	91.7	93.6	92.7		93.4	99.9
NC_025805.1_Cangyuan	Bat	92.3	91.9	91.4	91.5	93.7	93.8	82.4	90.7	82.8	81.8	90.8	92.4	91.1	93.4		93.6
ON548559.1_Pteropine_Malaysia	Human	93.8	93.6	93.1	93.0	98.9	96.2	82.9	91.8	83.6	82.0	91.8	93.7	92.9	99.9	93.6	

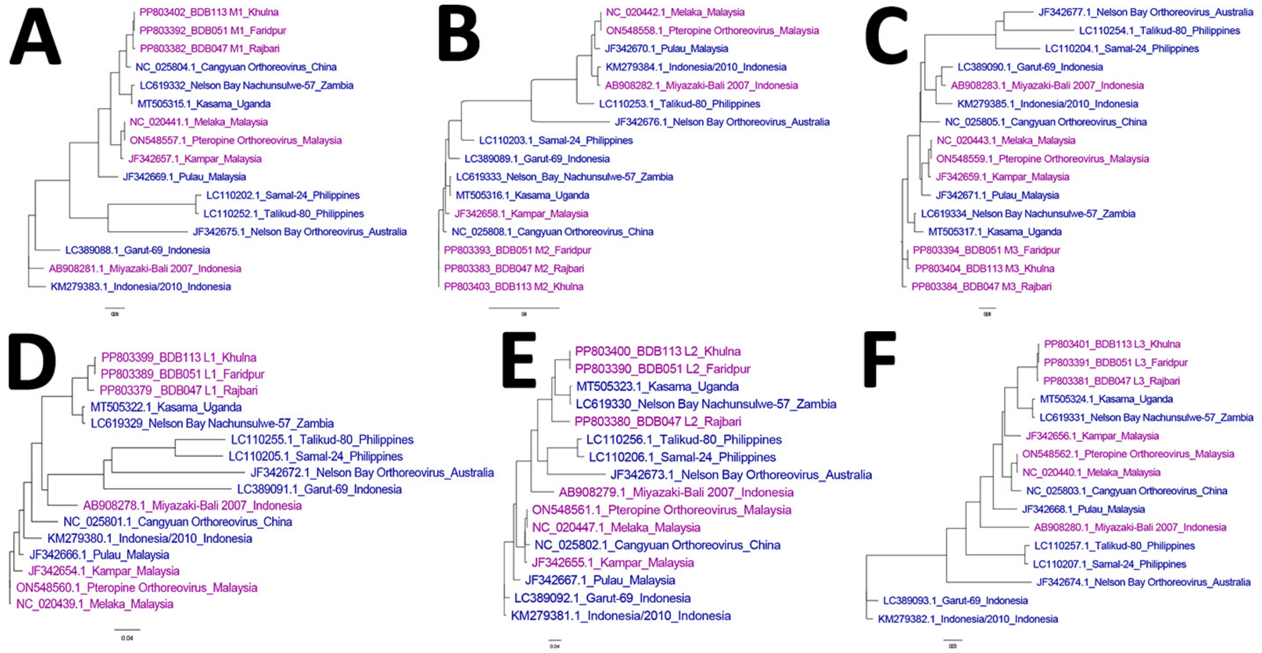

Appendix Figure. Phylogenetic analysis of the complete genomes of Pteropine orthoreovirus (PRV) isolates from Bangladesh. Six genome segments were analyzed: M1, M2, M3 (A–C); and L1, L2, L3 (D–F). The genomes of PRV isolates BDB047, BDB051, and BDB113 were retrieved using VirCapSeq-VERT analysis of cultured viruses sequenced on the Illumina platform. A global alignment of these segments was performed, including all comple te and partially sequenced PRV isolates from humans (violet font) and bats (blue font). Phylogenetic trees were constructed using IQ-TREE with model selection (GTR+G+I), and bootstrap support values (1,000 replicates) are shown next to each branching node. The scale bar represents nucleotide substitutions per site.
